# Structure of FlgK reveals the divergence of the bacterial Hook-Filament Junction of *Campylobacter*

**DOI:** 10.1038/s41598-017-15837-0

**Published:** 2017-11-16

**Authors:** Paula V. Bulieris, Nausad H. Shaikh, Peter L. Freddolino, Fadel A. Samatey

**Affiliations:** 1 Trans-membrane Trafficking Unit, Okinawa Institute of Science and Technology Graduate University, 1919-1 Tancha, Onna, Kunigami, Okinawa 904-0495 Japan; 2 Department of Biological Chemistry, University of Michigan Medical School, Ann Arbor, Michigan USA

## Abstract

Evolution of a nano-machine consisting of multiple parts, each with a specific function, is a complex process. A change in one part should eventually result in changes in other parts, if the overall function is to be conserved. In bacterial flagella, the filament and the hook have distinct functions and their respective proteins, FliC and FlgE, have different three-dimensional structures. The filament functions as a helical propeller and the hook as a flexible universal joint. Two proteins, FlgK and FlgL, assure a smooth connectivity between the hook and the filament. Here we show that, in *Campylobacter*, the 3D structure of FlgK differs from that of its orthologs in *Salmonella* and *Burkholderia*, whose structures have previously been solved. Docking the model of the FlgK junction onto the structure of the *Campylobacter* hook provides some clues about its divergence. These data suggest how evolutionary pressure to adapt to structural constraints, due to the structure of *Campylobacter* hook, causes divergence of one element of a supra-molecular complex in order to maintain the function of the entire flagellar assembly.

## Introduction

Gram-positive and gram-negative bacteria use flagella to swim^1^, but in many taxa, flagella are also involved in causing infections^2^. In addition to conferring motility, the *Campylobacter jejuni* (*C. jejuni*) flagellum has the ability to export non-flagellar proteins with significant roles in *Campylobacter* virulence^3,4^. Moreover, the *C. jejuni* flagellum impacts cellular division^5^. The bacterial flagellum has a rotating motor located in the cell^6^ and an extra-cellular component that is formed of the rod, the hook and the filament^7^. In most bacteria, the filament, a fairly rigid structure that plays the role of a propeller, comprises an assembly of about ten thousand copies of a single protein, FliC. About a hundred copies of another protein, FlgE, make the hook. The hook is a very flexible structure and it serves as a universal joint^8^. A special junction to assure a smooth connectivity is needed to link the hook and the filament, which have different structural and functional characteristics. This junction is composed of two ring-like structures made of multiple copies of FlgK and FlgL, respectively^9^ (Fig. S1). In this junction, the first segment, composed of the FlgK complex, is in contact with the hook, while the second segment, made by the FlgL complex, connects to the filament^9^. Here, we solved the three-dimensional structure of a major fragment of FlgK from *C. jejuni*, strain ATCC 700819/NCTC 11168, using X-ray crystallography. Based on this structure, a model of the FlgK ring was made and fitted onto the model of the *Campylobacter* hook. The structure of FlgKcj58 reveals that the cell-proximal half of the protein, which includes the N- and C- termini, and which is known to connect to the hook^9^, is structurally conserved when compared with other known FlgK protein structures. However, the cell-distal half of the protein has diverged, developing a different 3D structure.

## Structure of FlgK from ***C. jejuni***

**Figure 1:**
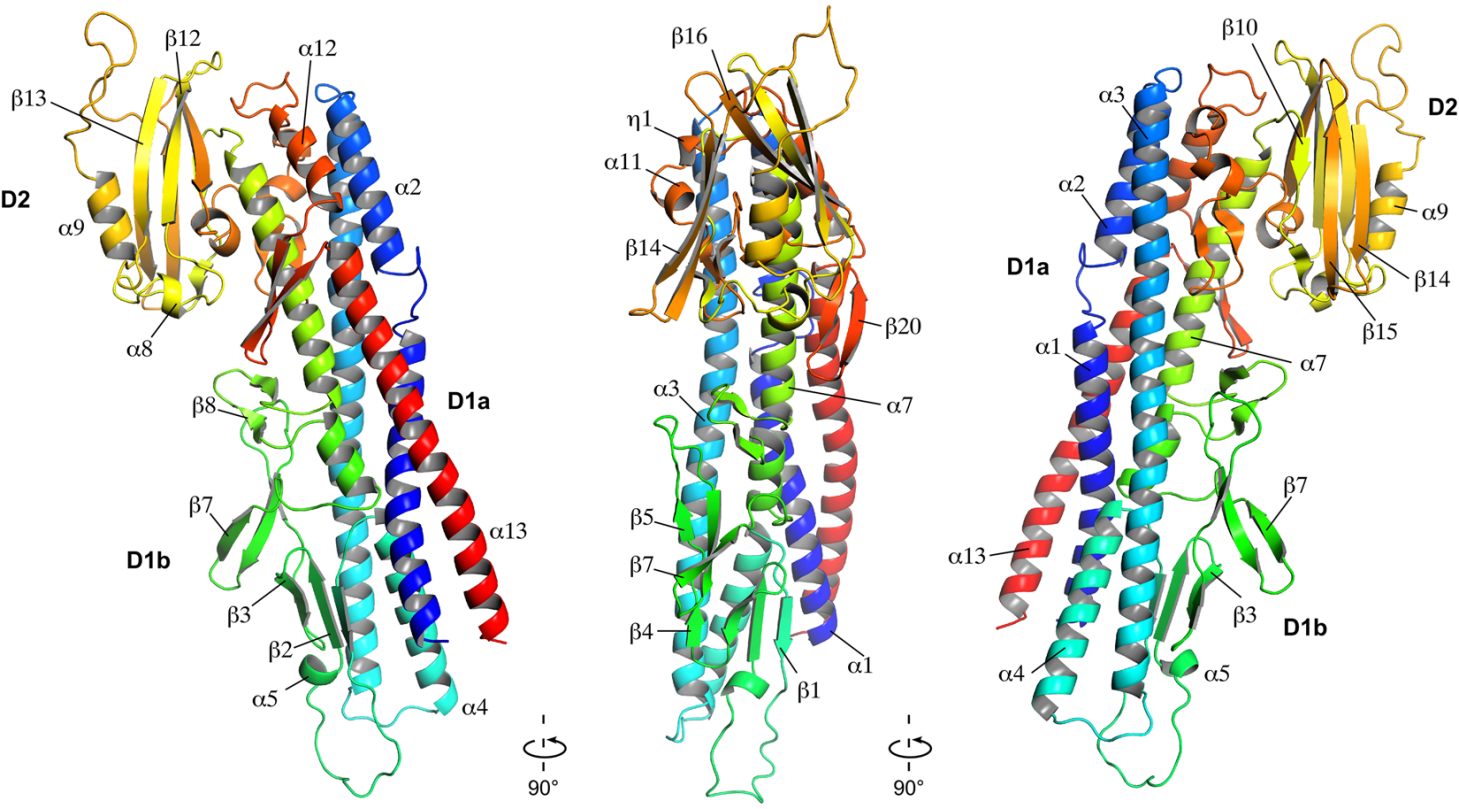
Structure of FlgK protein from *C. jejuni*. View of the Cα backbone trace of FlgKcj58. The chain is colour coded from blue to red from the N to the C terminus. FlgKcj58 has two domains, D1 and D2, with D1 divided into two sub-domains, D1a and D1b. Residues are labeled with one-letter codes as a guide to follow the chain. (Figure prepared with PyMOL, The PyMOL Molecular Graphic System, Schrödinger, LLC., http://www.schrodinger.com/suites/pymol).

The proteins that form the extra-cellular part of the bacterial flagellum polymerize by interaction of their N- and C-termini, which are disordered in solution^10,11^. To crystallize FlgK (67 kDa) of *C. jejuni* strain ATCC 700819/NCTC 11168, we removed 68 and 28 amino acid residues from the N- and C- termini, respectively. The remaining 58 kD fragment, named FlgKcj58, has 511 amino acids (See Methods for detailed experimental procedures). The crystal belongs to the orthorhombic space group *P*2_1_2_1_2 and it diffracted to 2.45 Å resolution (Table 1). The structure of FlgKcj58, in which 11 C-terminal residues were not resolved, comprises the fragment [Asp69-Gly568]. FlgKcj58 can be described as composed of two domains. There is a central domain, D1, which could be divided into two sub domains, D1a and D1b, and a more compact domain D2, consisting mostly of β-strands (Fig. 1). Sub-domain D1a, which has eight discontinuous α-helices, could be described as a 4-helix bundle. The first segment of the helical bundle of domain D1, in the N-terminal region, is composed of α-helices α1 and α2, which consist of segments [Asp69-Arg99] and [Ile108-Asn124] that are connected by a short coil. The second segment of the bundle is made from a single long helix α3 that runs through the length of domain D1a. The third segment of the bundle is composed of 3 tandem helices, α4, α6 and α7 that consist of segments [Thr193-Leu213], [Gln291-Arg299] and [Ile314-Ser339], respectively. Helices α4 and α6 are separated by the sub-domain D1b. The fourth segment of the bundle consists of α-helices α12 and α13, which correspond to segments [Asp500-Tyr510] and [Met527-Gly568], respectively. These two helices are separated by a β-hairpin β19-β20. Domain D1b, which consists of segment [Val214-Gly289], is made of 7 antiparallel β-strands, β1 to β4 and β6to β7, connected by relatively short loops. Domain D2 comprises segment [Ser340- Asn499]. It is located at one extremity of the helical bundle of domain D1 and consists of a combination of antiparallel β-strands connected by small loops and α-helices.

**Table 1:** Summary of crystallographic data for FlgKcj58 crystals.

Data collection		Refinement	
Wavelength	0.9	Resolution (Å)	19.95–2.45 (2.536–2.45)
Total reflections	293378	No. reflections	21523 (2063)
Space group	*P*2_1_2_1_2	R_work_	0.1811 (0.2190)
Cell dimensions (Å)		R_free_	0.2334 (0.2908)
*a*	49.50	Non-hydrogen atoms	4322
*b*	123.55	Protein residues	500
*c*	92.54	Water molecules	399
Resolution (Å)	40.0–2.45 (2.49–2.45)	*B*-factors	28.83
I/σ(I)	12.8 (3.3)	Protein	28.957
*R*_merge_	0.18 (0.7)	Water	31.42
		R.m.s deviations	
Completeness (%)	100.0 (99.7)	Bond length (Å)	0.003
		Bond angle (°)	0.55
Redundancy	7.3	Ramachandran plot (%)	
		Favored	96.2
		Allowed	3.4
		Outliers	0.4

^†^ *R*_merge_ = ∑_*hkl*_ ∑_*i*_|*I*_*i*_(*hkl*) − 〈*I*(*hkl*)〉|/∑_*hkl*_ ∑_*i*_ *I*_*i*_(*hkl*), where *I*_*i*_(*hkl*) is intensity of the *i*th measurement of reflection *hkl* and 〈*I*(*hkl*)〉 is the mean value of *I*_*i*_(*hkl*) for all *i* measurements.

## Divergence of FlgK from ***C. jejuni***

**Figure 2:**
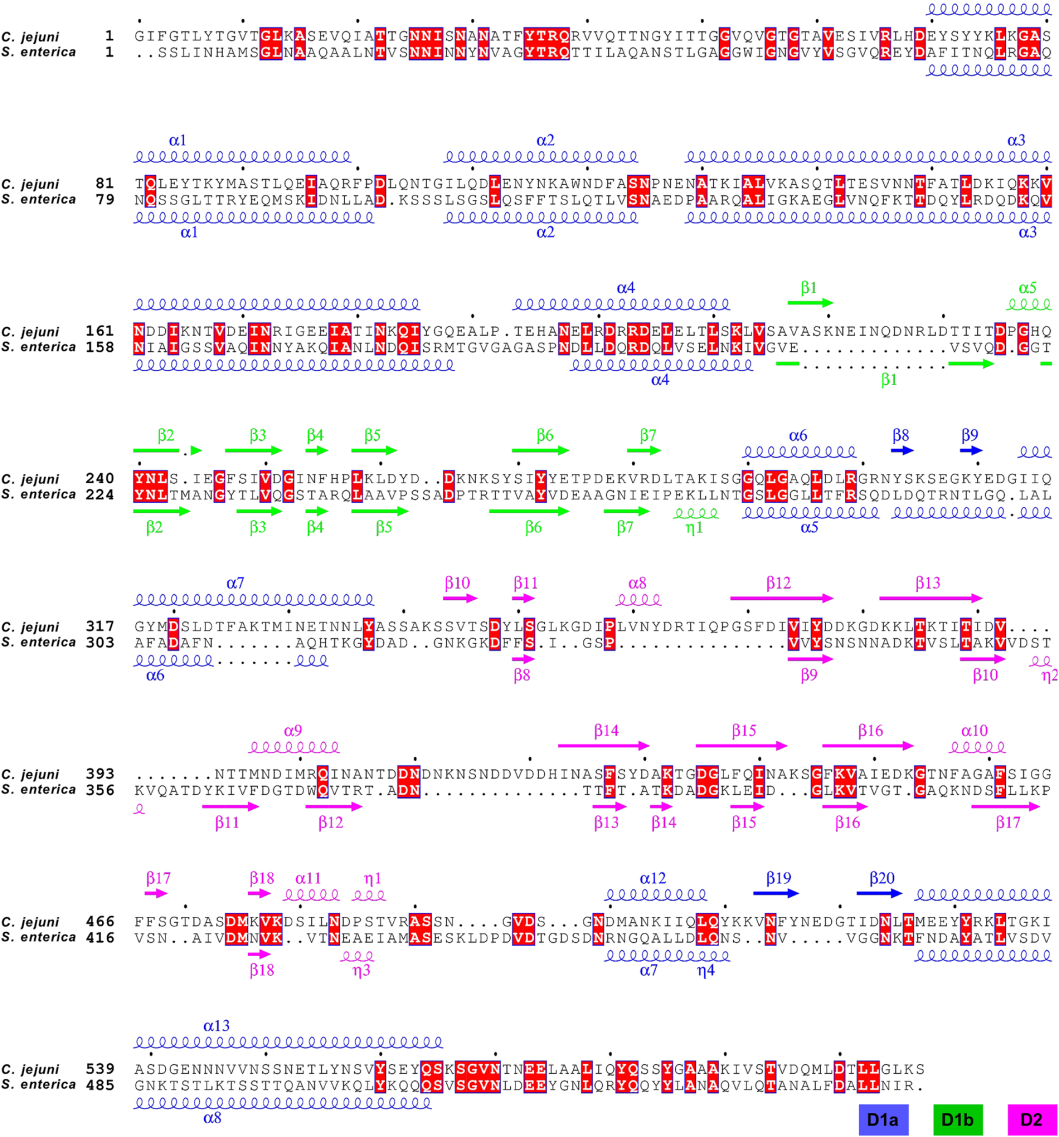
Sequence alignment of FlgK proteins. Sequence alignment of FlgK from *C. jejuni* and *S. enterica* with a representation of their respective secondary structure. Domain D0, composed of the N-terminal and the C-terminal chains, which are involved in coiled-coil interactions, was missing from each of these structures obtained by X-ray crystallography. The secondary structure of domains D1a, D1b, and D2, found in FlgKcj and FlgKse, are in blue, green, and purple, respectively. The red squares represent the conserved amino acid residues between both sequences. Amino acid sequences of proteins were aligned using Clustal Omega^37^, and secondary structure rendering used ESPript 3.0 (ref.^38^).

**Figure 3:**
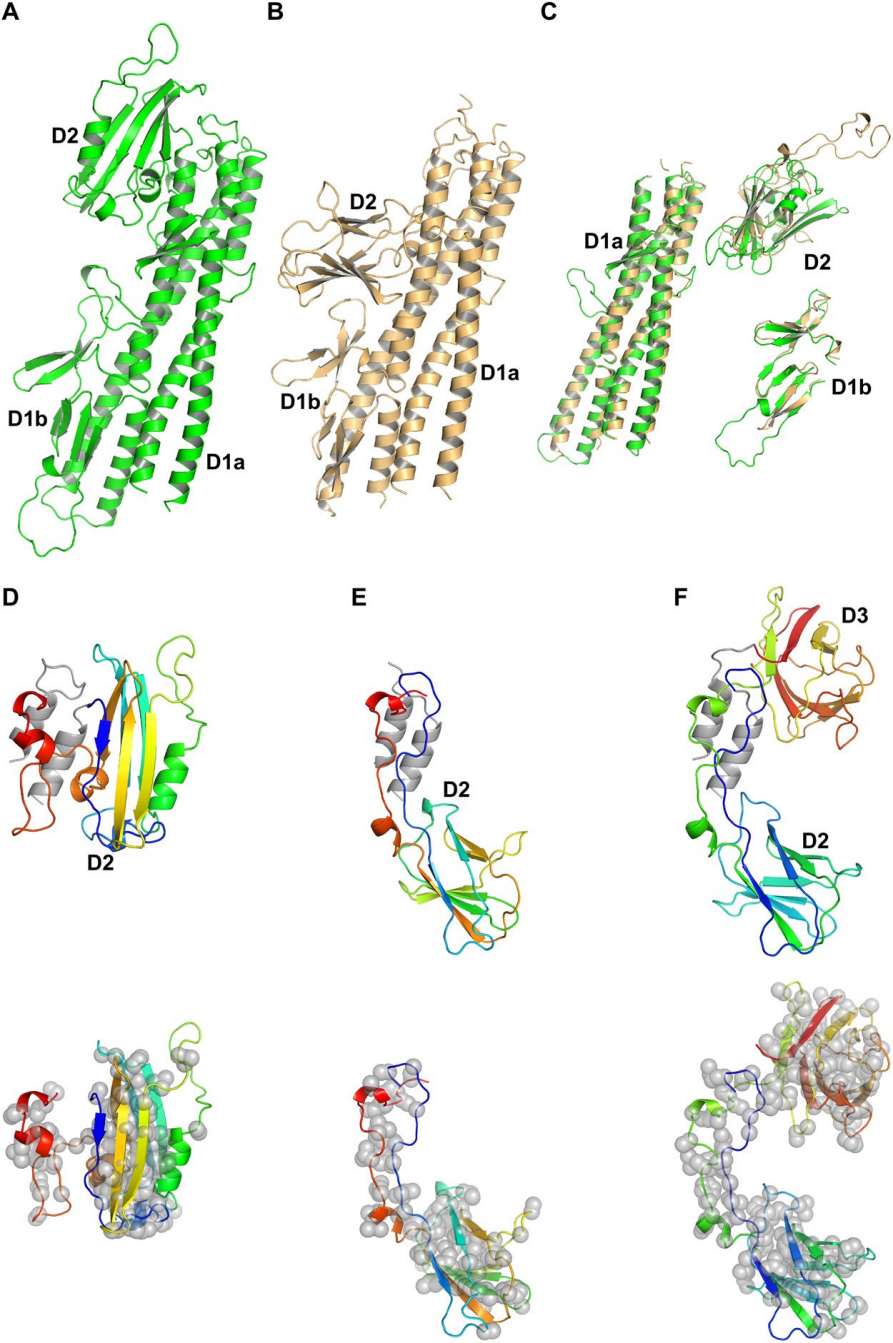
Structural comparison of Flgk from *C. jejuni* and *S. enterica*. A Cα backbone trace of FlgKcj58 in green (**A**) and FlgKse49 in gold (**B)**. Both structures have the same number of domains but domains D2 have a different folding. (**C**) Superposition of FlgKcj58 and FlgKse49 domains D1a, D1b, and D2, respectively. Domains D1a superimpose with an RMSD 1.2 Å over 213 residues. Domains D1b superimpose with an RMSD 1.4 Å over 48 residues. Domains D2 superimpose with an RMSD 1.8 Å over 47 residues. Domains D1a from FlgKcj58 and FlgKse have 270 and 246 residues, respectively. Domains D1b from FlgKcj58 and FlgKse have 75 and 60 residues, respectively. Domains D2 from FlgKcj58 and FlgKse49 have 160 and 120 residues, respectively. The alignments were done with C-alpha Match^39^. Cα backbone trace of domains D2 from FlgKcj58 (**D**), FlgKse49 (**E**) and FlgKbp64 (**F**) showing their respective position to domain D1a α-helices in Gray. The relative position of domain D2 of FlgKcj58 differs from that of FlgKse49 and FLgKbp64. Lower panel shows the distribution of hydrophobic residues side chains, represented by gray spheres, superimposed on a cartoon representation of domain D2 of FlgKcj58 (**D**), FlgKse49 (**E**) and FlgKbp64 (**F**). For FlgKcj38 (**D**) and FlgKse49 (**E**), domain D2 is colour-coded from the N-terminal chain (blue) to the C-terminal chain (red). FlgKbl64 (**F**), which has a third domain connected to its domain D2, domain D2-D3 is colour-coded from the N-terminal chain (blue) to the C-terminal chain (red). (Figure prepared with PyMOL, The PyMOL Molecular Graphic System, Schrödinger, LLC. http://www.schrodinger.com/suites/pymol).

Previously, structures of two orthologous proteins of FlgKcj have been solved by X-ray crystallography. The structure of a 64 kDa fragment of FlgK from the beta-proteobacterium *Burkholderia pseudomallei* (*B. pseudomallei*) has been previously reported^12^. Furthermore, a 49 kDa fragment of FlgK from the gamma-proteobacterium, *Salmonella enterica typhimurium* (*S. enterica*) has been deposited in the Protein Data Bank (PDB-id 2D4Y). These two structures will be hereafter referred to as FlgKbp64 and FlgKse49, respectively. Structural comparison shows that FlgKcj58 has diverged from both of these structures. The full-length FlgK protein from *S. enterica* (FlgKse) is 59 kDa while FlgK from *B. pseudomallei* (FlgKbp) is 67.4 kDa. FlgKcj has sequence identities of 24% and of 21% to FlgKse and FlgKbp, respectively, and sequence similarities of 40% and 35%. Insertions in the sequence can be found at various positions (Fig. 2). For comparison, FlgKse and FlgKbp have a sequence identity and similarity of 32% and 48%, respectively. All three structures of FlgK, FlgKcj58, FlgKse49, and FlgKbp64, lack the N- and C- terminal segments that form the coiled-coil of domain D0, seen in the structures of the bacterial flagellar hook and filament^13,14^. The three-dimensional structure of FlgKcj58 aligned to that of FlgKse49 and of FlgKbp64 with a root mean square deviation (RMSD) for alpha carbons of 1.38 Å over 302 residues and 1.54 Å over 291 residues, respectively. Overall structural alignments show that domain D1 of FlgK is well conserved (Figs 3A–C, S2, S3).

The structure of the D1a domain of FlgKcj58, which consists of the helical bundle, is similar to that of FlgKse49 and of FlgKbp64. However, small differences can be noticed in this domain. The β-strands β10 and β20, which divide the C-terminal helix in FlgKcj58, is replaced by a short coil in both FlgKse49 and FlgKbp64. Furthermore, FlgKcj58 has an inserted segment [Gly300 - Gly313], which divides the segment [Gln291 - Ser339] into helices α6 and α7, while the equivalent segment corresponds to a continuous long α-helix in both FlgKse49 and FlgKbp64. The structure of domain D1b is conserved in all these three structures of FlgK.

Domain D2 of FlgKcj58 has a different fold compared to domains D2 of FlgKse49 and of FlgKbp64 (Figs 3A-C, S1,S3). Domain D2, in FlgKbp64 and FlgKse49, can be described as a beta-barrel made by eight β-strands connected by long loop. On the other hand, in FlgKcj58, the domain D2 has six β-strands, in a “V” shape, completed with α-helices. Furthermore, the relative position of domain D2 of *C. jejuni* FlgK differs from that of domains D2 of FlgK in both *S. enterica* and *B. pseudomallei*. FlgKbp64 has a third domain, D3, that neither FlgKse49 nor FlgKcj58 have. This domain D3 of FlgKbp64 has a fold similar to that of its domain D2 (Fig. S3). In FlgKcj58, a short loop links the N-terminus of domain D2 to the helix α7 in domain D1 (Fig. 3D). The size of this loop would prevent domain D2 of FlgKcj58 from adopting a similar position as in FlgKse49 or FlgKbp64. In the case of FlgKse49 and FlgKbp64, their D2 domain is linked to domain D1 by long loops (Fig. 3E,F). These long loops might have allowed a different position for domain D2 in the case of FlgKse and FlgKbp. However, in FlgKbp, domains D2 and D3 form a continuous chain and the position of domain D3 would prevent placing domain D2 in a similar position as in FlgKcj. Furthermore, the distribution of hydrophobic residues in the core of domain D2 (Fig. 3D–F) clearly shows that domains D2 and D3 form compact globular domains with little possibility for extensive displacement. These observations lead us to believe that the position of domain D2 is not an artifact due to crystallographic contacts. This position of D2 is close to what it would be in the flagellum, with of FlgK serving as a linker between the hook and FlgL.

## Model of the FlgK ring and the hook

**Figure 4:**
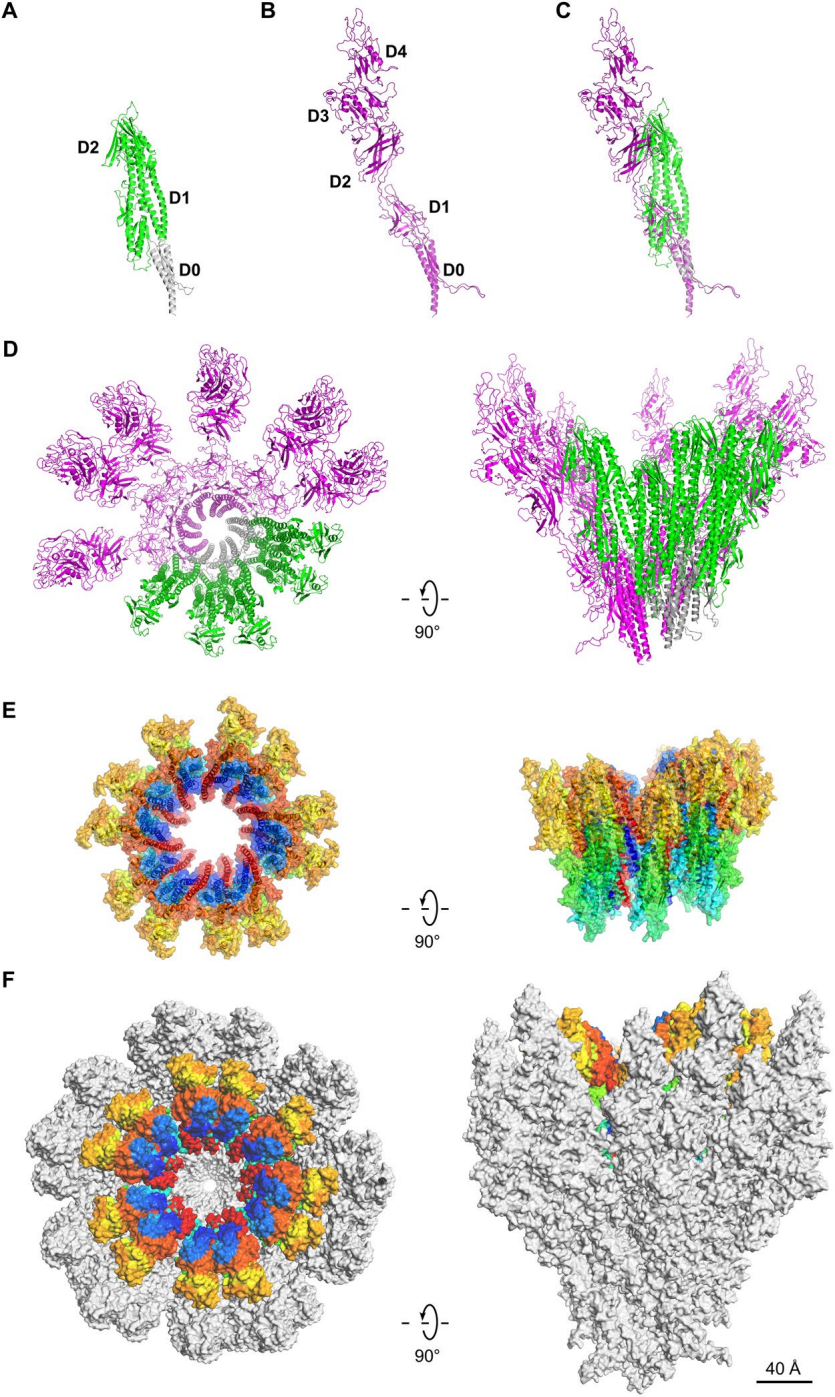
Structural alignment of FlgK and FlgE and Model of the FlgK ring with 11 molecules. (**A**) View of the complete structure of FlgK from *C. jejuni*. Domain D0, in gray, was obtained by homology modeling (template: 5JXL). Domains D1 and D2, in green, are the structure of FlgK58cj obtained by X-ray crystallography. (**B**) Structure of FlgE from *C. jejuni* (PDB id 5JXL). (**C**) Superposition of FlgK and FlgE from *C. jejuni* by aligning their D0 domains. (**D**) View from the distal end and lateral view of a ring of the hook with 4 molecules of FlgE substituted with 4 molecules of FlgK. FlgE molecules are in magenta. FlgK molecules are in green for the part corresponding to FlgKcj58 and in gray for the part corresponding to domain D0 that makes the central region together with domain D0 of FlgE. (**E**) View from the distal end and lateral view of the refined ring made of 11 molecules of FlgKcj58. Each molecule of FlgKcj58 is colour-coded in a spectral (‘rainbow’) sequence, from the N-terminus in blue to the C-terminus in red. (**F**) View from the distal end and lateral view the ring, made of FlgKcj58, docked on top of the hook of *C. jejuni* which surface is colored in gray. (Figure prepared with PyMOL, The PyMOL Molecular Graphic System, Schrödinger, LLC. http://www.schrodinger.com/suites/pymol).

The proteins that comprise flagellar axial protein complexes, such as FliC for the filament, FlgE for the hook, and FlgG for the rod, frustrate crystallization of the full-length proteins because of their tendencies to polymerize or to aggregate in solution^9,15,16^. A domain D0, found in all these proteins, is involved in coiled-coil interactions, preventing crystallization of full-length proteins, so crystallization is only possible after removal of this domain^17,18^. Domain D0 is missing from all the X-ray crystallography structures of FlgKcj58, FlgKbp67 and FlgKse49. A model of the full-length FlgK was built using Swiss-Model^19,20^, which selected the hook protein of *Campylobacter* ^13^ (FlgEcj, PDB-id 5JXL) as the best template for domain D0 of FlgK. Domains D0 of FlgKcj and FlgEcj share 33% sequence identity and 48% sequence similarity. For comparison, domains D0 of FlgEcj and of the distal rod protein FlgG from *Campylobacter*, which are known to have very similar structures, share 29% sequence identity and 45% sequence similarity. The resulting theoretical model includes the 3D structure of domain D0 of FlgKcj, not present in FlgKcj58 (Fig. 4A). Domain D0 of FlgKcj was first aligned to domain D0 of the FlgE protein in the hook (Fig. 4B,C), enabling the construction of a ring made of eleven molecules of FlgKcj58; we then performed further structural refinement as described under Methods (Fig. 4D,E). The overall structure of the bacterial flagellar hook and filament consist of a structure made of eleven protofilaments with the N and C terminal chains being the driving force of the structural organization through coiled-coil interactions^8,13,16^. The high sequence similarity between D0 domains of FlgKcj and FlgEcj makes us to believe that the ring of FlgK will also consist of eleven molecules, each interacting with one molecule of FlgE in the hook. The ring was docked on top of the hook of *C. jejuni*, initially using domain D0 of FlgE as a template for alignment (Fig. 4F; see Methods for details). When docked onto the structure of the *Campylobacter* hook, the FlgK ring is almost completely hidden by molecules of the hook, leaving only the tips of a few molecules of FlgKcj protruding from the top of the hook (Fig. 4F).

In the bacterial flagellum, the lengths of the hook and the rod are well controlled^21,22^, but the mechanism controlling the number of FlgK molecules necessary to make the junction between the hook and the filament is unknown. However, a model of this junction, consisting of more than eleven molecules of FlgK causes clashes between domains D1a of the hook-distal FlgK molecule with the hook-proximal domains. These steric constraints seem to limit the number of FlgK molecules to eleven.

## Discussion

*Campylobacter* is the only known bacterium that secretes toxins through its flagella. Understanding the overall structure of the flagellum of *C. jejuni* is important. The model of the FlgK ring from *C. jejuni* and the model of the connection between the ring and the hook from *C. jejuni* give some clues about why the structure of FlgKcj has a different folding compared to those of FlgKse and FlgKbp.

**Figure 5:**
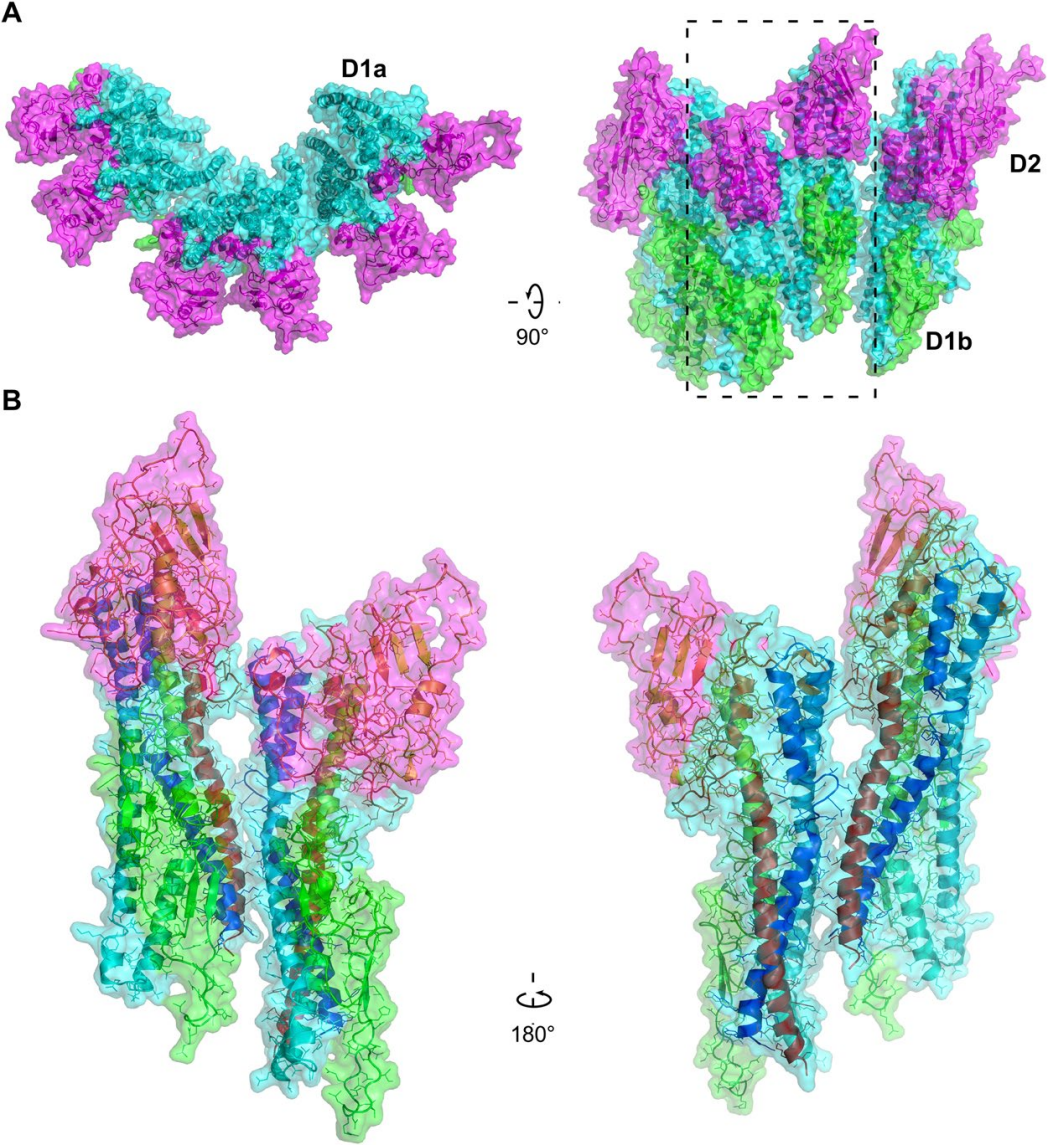
Molecular interactions within the molecules in FlgKcj58 ring made of eleven molecules. (**A**) View from the distal end and lateral view of six of FlgKcj58. Domain D1a, which makes the inner part of the ring, is in cyan. Domain D1b and D2, which make the external surface of the ring, are colored in green and magenta, respectively. (**B**) Close-up view of the boxed region in (**A**) showing two molecules of FlgKcj58 as they appear in the FlgK ring. Cα backbone trace of each molecule is color-coded from blue to red from the N to the C terminus, while the surface is colored in cyan, green and magenta for domain D1a, D1b and D2, respectively.

The ring made of FlgKcj58 was refined prior to its docking on the top of the hook molecule. The refined model of the ring shows eleven well packed molecules of FlgKcj58 with no room for extra molecules. FlgKcj58 from the refined structure has an RMSD of 3.3 Å with the crystal structure. The obtained FlgK ring has a diameter of 200 Å (Fig. 5A). In this model, domains D1b and D2 make the external surface of the ring while helices of domain D1a make the central core. Most interactions within the molecules in the ring, are the between domain D1a of neighboring molecules (Fig. 5B) between the beginning of α-helix α1 and the end of α-helix α13. We also found some interactions between the short helix α11, in domain D2, and the β20 that divides helices α12 and α13 in domain D1a. The subtle interactions are not surprising as most of the interactions will be between domains D0, similar to the interactions seen between FlgE molecules in the hook^13^.

**Figure 6:**
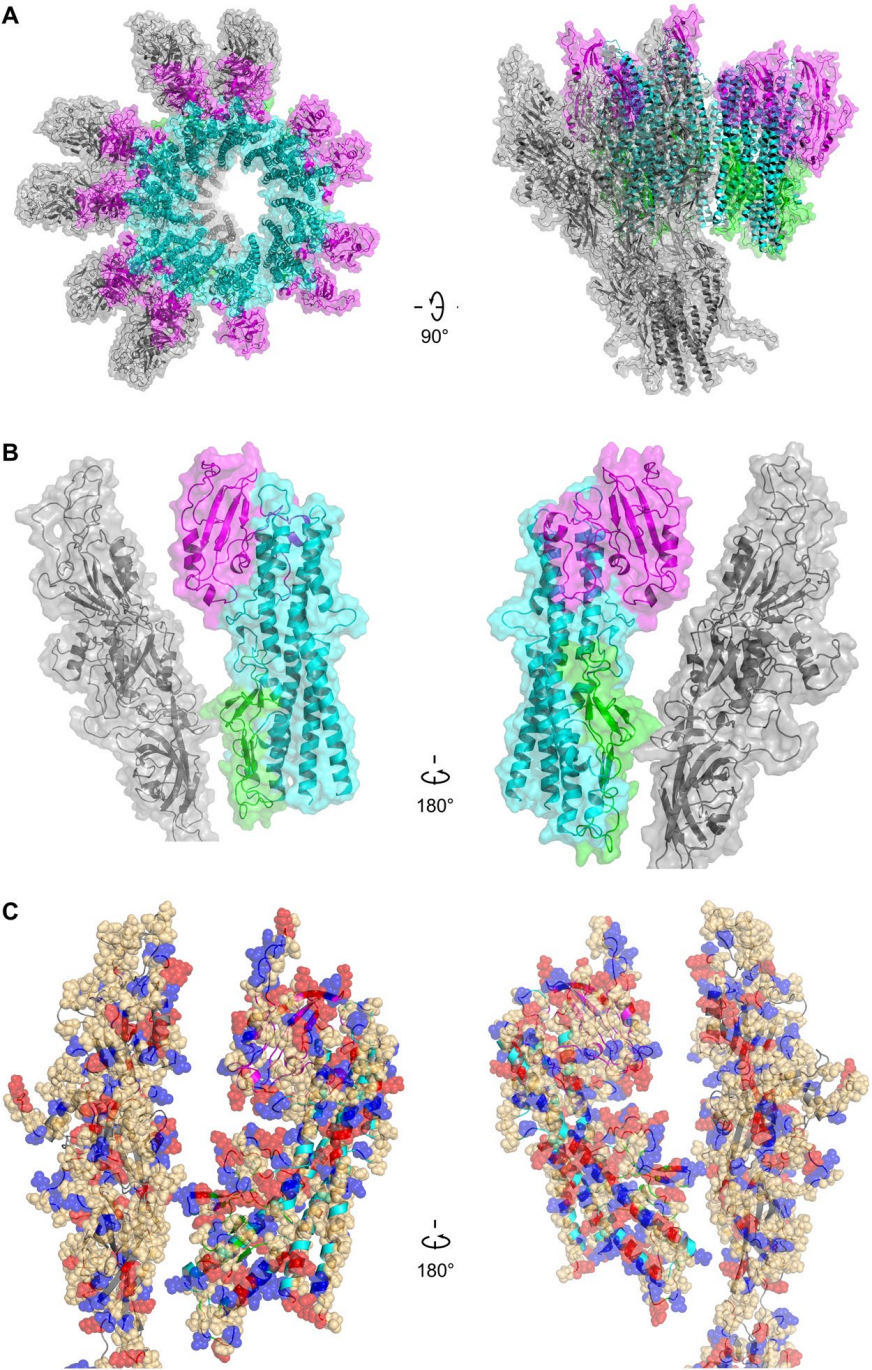
Refined model of the molecular junction between the hook and FlgKcj58 ring. (**A**) View from the distal end and lateral view showing the Cα backbone trace of six molecules of FlgE in gray with the ring of FlgKcj58 with domains D1a, D1b and D2 in cyan, green and magenta, respectively. (**B**) View of the interactions between FlgKcj58 and FlgE taken from the Hook-FlgKcj58 junction: domains D1a, D1b and D2 are colored in in cyan, green and magenta, respectively, while FlgE is in gray. (**C**) Distribution of charged and polar residues in the interactions between FlgKcj58 and FlgE taken from the Hook-FlgKcj58 junction: positively charged (Arg, Lys, His), red; negatively charge (Asp, Glu), blue; polar (Ser, Thr, Asn, Gln), light orange.

The junction between the hook and FlgKcj58 ring was refined by optimizing the relative orientation and position of the FlgKcj ring (see Methods for details). The refined junction shows that FlgE molecules in the hook interact mostly with domain D1b of FlgKcj58 (Fig. 6). Domain D0, which is missing from FlgKcj58, would make the contacts to assure the continuation of the core region made by domain D0 of FlgE and FlgK (Fig. 4D). Domain D2, which does not seem to have any contacts with FlgE, might serve as the interacting domain in the FlgK-FlgL ring junction.

**Figure 7:**
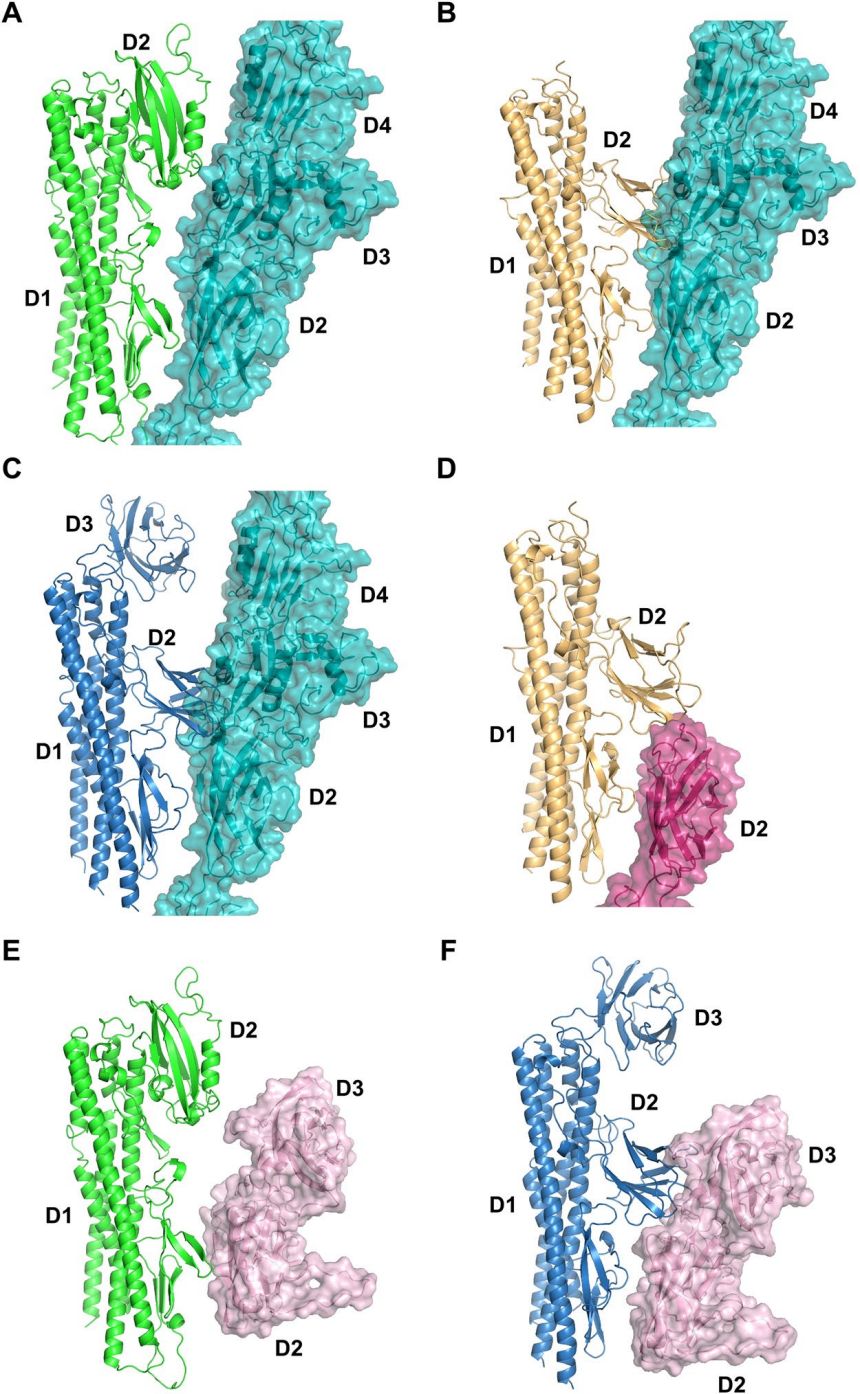
Interaction between molecules of FlgK and FlgE in FlgK-Hook docking configurations. Comparison of the interaction between FlgK and FlgE molecules from the hook in different configurations: (**A**) Interaction between FlgKcj58, in green, and FlgE from *C. jejuni* in cyan. (**B**) For comparison, interactions between FlgKse49 in gold and FlgE from *C. jejuni* in cyan showing the clashes between domain D2 of FlgKse49 and domain D3 of FlgE from *C. jejuni*. (**C**) For comparison, interactions between FlgKbp64 and FlgE from *C. jejuni* with the clashes between domain D2 of FlgKbp64 and domain D3 of FlgE from *C. jejuni*. (**D**) Interaction between FlgKse49 and FlgE molecule from *S. enterica* in dark pink. Interaction FlgEcj58 in green (**E**) and FlgEbp64 in dark blue (**F**) with FlgE from *C. crescentus* in light pink.

The necessity for divergence of the structure of *Campylobacter* FlgK arises, in part, from the structure of its hook protein, FlgE. *Campylobacter* FlgE (FlgEcj) has two extra domains compared to *S. enterica* FlgE (FlgEse)^13,23^. The model of the junction between FlgKcj and the hook shows that sub-domain D1b of FlgKcj, which is conserved in all known structures of FlgK, interacts with domain D2 of FlgE. Structural studies of FlgE from different proteobacteria have shown that domains D0, D1, and D2 of FlgE are structurally well conserved^13,23–25^. Based on structural homology of the FlgK and FlgE proteins, it is fair to assume that sub-domain D1b of FlgK will interact with domain D2 of FlgE as shown in our model. Our model of the junction shows that domain D2 of FlgKcj58 interacts with domains D3 and D4 of FlgEcj from *C. jejuni* (Fig. 7A). Structural alignment of FlgKcj and FlgKse shows that, if FlgKcj had a 3D folding similar to that of FlgKse, with its domain D2 located in the same position as for FlgKse49, its domain D2 would prevent formation of the junction with FlgEcj due to steric clashes (Fig. 7B). Indeed, domains D2 of FlgKse and FlgKbp would superimpose to domain D3 of FlgEcj (Fig. 7C). In the case of *S. enterica*, domain D1b of FlgKse would interact with domain D2 of FlgE from *S. enterica*, while domain D2 of FlgKse would interact with the top part of domain D2 of FlgEse (Fig. 7D) and eventually makes contact with the ring of FlgL, which connect FlgK to the filament (Fig. S1).

The hook protein of *Caulobacter crescentus* (*C. crescentus*), FlgEcc, which structure has previously been reported^25^, has an extra domain D3 not found in *S. enterica*. However, Yoon and colleagues have shown that domain D3 from FlgEcj and domain D3 of FlgEcc have different structures and are positioned differently compared to the other domains^25^. It would be interesting to know how our proposed models apply to the hook of *C. crescentus*. With 702 amino acid residues, the sequence of FlgK from *C. crescentus* strain CB15 (ATCC 19089) is longer than the sequence FlgK previously described. The sequence comparison of FlgK protein from *C. jejuni*, *S. enterica*, *B. pseudomallei* and *C. crescentus* shows that, like FlgKbp, FlgKcc has third domain D3 (Fig. S4). FlgKcc has a sequence identity of 23.7% and a sequence similarity of 37% with FlgKbp, which is higher than with FlgKse and FlgKcj. This makes us to believe that the structure *C. crescentus* FlgK, which is not known, might be similar to that of *B. pseudomallei*, with a slightly larger domain D3 (Fig. S4). Our FlgE-FlgK junction model shows that domain D2 of FlgKcj could not interact with domain D3 of FlgEcc (Fig. 7E), making it a possible fit as a junction for *C. crescentus* hook. However, FlgKbp could be a better model for FlgK of *C. crescentus* (Fig. 7F) and the sequence alignment of FlgKcc and FlgKbp (Fig. S4) seems to support this hypothesis.

Bacterial flagella grow in a sequential process^26,27^. Once the hook is completed, FlgK starts appearing at the top of the hook to form the first junction, followed by FlgL that builds up at the top of the ring made of FlgK. The hook is very flexible, while the filament, although more rigid, is able to undergo structural transitions between different states with distinct helical properties^14,28^. The junctions made by FlgK and FlgL play important roles in bridging the hook to the filament. The structural divergence of FlgKcj is in accordance with the existence of extra domains in the hook of *C. jejuni*, but pressure for this change could have diverse origins. In *Campylobacter* and in related organisms, FlgL, has between 750 and 950 residues compared to 410 for *Burkholderia* and 317 for *Salmonella*. The divergence of FlgKcj is also an indication of the changes that could be expected in the connection between FlgK and FlgL in *Campylobacter*. Overall, the structural divergence of FlgK from *Campylobacter* is a case where one element of a supra-molecular complex diverges to compensate for changes in another element, in order to maintain the entire assembly and its function.

## Methods

### Cloning

The DNA sequence encoding FlgK57cj (amino-acid residues 70–580) was amplified by PCR from *C. jejuni* strain NTCT11168 genomic DNA with the 5′primer GGCTCTCATATGGATGAGTATTCTTACTATAAATTAAAAGGTGC, generating an NdeI site and a start codon, and the 3′ primer GCGACGCTCGAGATTATATAAGGGCGGCTAATTCTTCATTTGTAT, generating a stop codon and an XhoI site. The PCR fragment was digested with NdeI and XhoI and ligated into the T7 expression vector pET22b (+) (Novagen). The plasmid was transformed into *Escherichia coli* strain BL21 (DE3) for expression.

### Protein expression and purification

BL21 (DE3) cells harboring FlgKcj58 were grown in 6 L Luria broth (LB) containing antibiotic 100 μg mL^−1^ ampicillin. Protein expression was induced with 1 m*M* isopropyl β-D-1-thiogalactopyranoside (IPTG) at an OD_600_ of 0.8 and cultivation continued for 4 h at 310 K. The cell culture broth was harvested by centrifugation at 8000 *g* for 15 min. The cell pellet was suspended in 20 m*M* NaCl, 50 m*M* Tris-HCl pH 8.0 buffer and cells were disrupted by sonication. The solution of sonicated cells was then centrifuged at 100 000 *g* for 1 h and the supernatant was loaded onto a HiLoad Q Sepharose ‘High Performance’ anion-exchange column (GE Healthcare) equilibrated with 20 m*M* NaCl, 50 m*M* Tris-HCl pH 8.0 buffer. Protein elution was performed with a linear gradient of NaCl from 0 to 0.5 *M*. The main fractions were dialyzed overnight against 50 m*M* Tris-HCl pH 8.0 buffer and loaded again onto the same Q Sepharose column. The anion exchange chromatography procedure followed by dialysis was repeated three more times in order to bring the protein to an optimal level of purity. Then eluted fractions were pooled and loaded onto a Superdex 200 gel-filtration column (GE Healthcare) equilibrated in 100 m*M* NaCl, 10 m*M* Tris-HCl pH 8.0 buffer and eluted with the same solution. Gel filtration fractions were pooled and dialyzed overnight against 5 m*M* Tris-HCl pH 8.0 buffer. The protein was concentrated to 16 mg ml^−1^ using a Centriprep centrifugal filter device (Millipore). The protein stock solution was stored at 277 K.

### Crystallization and data collection

The initial screening of crystallization conditions was carried out using the sitting drop vapour-diffusion method (200 nL protein solution was mixed with 200 nL reservoir solution and equilibrated against 120 μL reservoir solution) in 96-well plates using an automated nanolitre liquid-handling system (Mosquito, TTP labtech). The following screening kits were employed: Wizard I, II, III, and Cryo I, II (Emerald Biosystems) and Crystal Screen (Hampton Research). Two different protein concentrations: 7 and 16 mg mL^−1^ were used and crystallization plates were equilibrated at 293, 288, 283 and 278 K. Crystals of FlgKcj58 were obtained after 14 days, at 293 K, 16 mg mL^−1^ with condition 9 of Crystal Screen I (30% PEG 4k, 0.2 M ammonium acetate, 0.1 M sodium citrate pH 5.6). Optimization of FlgKcj58 crystals was performed manually, using the hanging-drop vapor diffusion method with a reservoir volume of 1 mL and a drop volume of 4 μL. The optimal crystallization buffer was found to be: 30% PEG MME 2,000, 0.4 M ammonium acetate, 0.1 M sodium citrate pH 5.6, 2% MPD, 4% 2-propanol and 5% ethylene glycol. Prior to X-ray diffraction measurements, all crystals were cryo-protected by soaking them briefly in a solution corresponding to the reservoir solution supplemented with 25% of ethylene glycol. Crystals were then mounted on a cryo-loop and data were collected at 100 K. Crystals diffracted at a resolution of 2.5 Å and belonged to the space group *P*2_1_2_1_2.

### Docking methodology

A model of the full-length FlgK protein was built using Swiss-Model^19,20^. This theoretical model included the 3D structure of FlgKcj domain D0 that is missing from the experimental structure of FlgKcj58 and was modeled based on FlgE of *Campylobacter jejuni* (PDB-id 5JXL). This model was used to build the initial ring of FlgKcj based on the alignment with FlgE molecule in the hook.

### Modeling of FlgK 11-mer

We generated an initial full-length FlgK structure by beginning with the D1D2 crystral structure, and then manually threading secondary structure elements from the D0 domain of FlgE onto regions with matching secondary structure predictions (via the I-TASSER server^29^) for D0. After refining the hybrid model using iterative steps of FG-MD^30^ and manual refinement to minimize clashes, we then threaded the complete FlgK sequence onto that model using I-TASSER to generate a complete monomer structure.

An initial structure for the FlgKcj ring (11-mer) was obtained based on the alignment with the hook molecule FlgE. We then performed minimal manual adjustments of the structure to remove clashes, and allowed the structure to relax in a molecular dynamics simulation by performing 5,000 steps of minimization and then 100 ps of Langevin dynamics at 298 K. Simulations were performed in NAMD 2.12 (ref.^31^) using the charmm36 protein force field^32^ and Generalized Born implicit solvent^33^ with default settings. We used a 1 fs timestep with 1.6 nm nonbonded interaction cutoff, with switching beginning at 1.5 nm, and with the strength of very close interactions clamped at 0.2 nm to avoid especially severe clashes. In addition, we imposed harmonic restraints on the backbone dihedral angles to keep them close to the values in the original I-TASSER structure, with a force constant of 5 kcal/mol. As a final refinement, we performed a fastrelax calculation on the final structure from the molecular dynamics trajectory using Rosetta (v2016.46.59086) (refs^34,35^). During the Rosetta relaxation calculation we restrained the backbone atoms to their starting coordinates, with a decreasing force constant and initial stdev parameter of 4.0.

### Modeling of FlgK-FlgE Interface

To model the interaction of our final FlgK 11-mer structure with a part of a FlgE, we initially placed the FlgK structure concentric with and above six FlgE molecules taken from the hook of *Campylobacter jejuni* (PDB-id 5JXL). We optimized the relative orientation of the FlgK ring by sampling rotations at 3 degree intervals and taking the rotation that minimized clashes between FlgK and FlgE monomers, and then raised the FlgK ring relative to FlgE until the majority of clashes were removed (implemented using TCL scripts in VMD 1.9.3 (ref.^36^)). To optimize the interaction distance between the FlgE and FlgK rings, we then performed three stages of molecular dynamics simulations, with all parameters as listed above except for omission of the backbone dihedral restraints. First, we performed 5,000 steps of minimization and 100 ps of Langevin dynamics; next, we applied 500 ps of steered molecular dynamics simulation (after an additional 500 step minimization to account for the new restraints) pulling the center of mass of the FlgK ring toward the FlgE, with harmonic restraints keeping the FlgE monomers in place, at a force constant of 500 kcal/(mol nm^2^). The pulling velocity was 0.01 nm/ps, with a spring constant of 1000 kcal/(mol nm^2^) in the pulling direction and 5000 kcal/(mol nm^2^) transverse to the pulling direction. After 500 ps of pulling, we analyzed the internal alpha carbon RMSDs of the FlgK subunits to determine at which point they began to deform. The position of the FlgK ring relative to FlgE immediately before large internal deformations of the FlgK monomers began was taken to be the optimal interaction distance. Beginning from that structure, we applied a final MD simulation of 1,000 steps minimization and 100 ps Langevin MD with no applied restraints, and then performed final relaxation with Rosetta as described above for the FlgK ring. In this final relaxation step, the D0 domain of FlgK was removed to avoid speculation regarding the interface of the uncrystallized portion.

### Data Availability

Atomic coordinates of FlgKcj58 have been deposited in the Protein Data Bank under accession code 5XBJ. The refined models have been deposited in DataDryad (doi:10.5061/dryad.fv8h6) and will be made available to the public. The X-ray diffraction data were collected in Spring-8 (Harima, Japan) under the proposal numbers 2012B6739, 2013B6845, 2014A6901, 2014B6901, 2015A6501.

## Electronic supplementary material

Supplementary Information
