# Supplementary material for: Structure of FlgK reveals the divergence of the bacterial Hook-Filament Junction of Campylobacter
Source: Sci Rep. 2017 Nov 16;7:15743. doi: 10.1038/s41598-017-15837-0 (PMC5691160; doi:10.1038/s41598-017-15837-0)
Supplement: Supplementary file 1 — Supplementary Information [file 41598_2017_15837_MOESM1_ESM.pdf]

Structure of FlgK reveals the divergence of the bacterial Hook-Filament Junction of *Campylobacter*

Authors:

Paula V. Bulieris[1], Nausad H. Shaikh[1], Peter L. Freddolino[2] & Fadel A. Samatey[1,*]

Affiliation:

1) Trans-membrane Trafficking Unit, Okinawa Institute of Science and Technology Graduate University, 1919-1 Tancha, Onna, Kunigami, Okinawa 904-0495, Japan.

2) Department of Biological Chemistry, University of Michigan Medical School, Ann Arbor, Michigan, USA

*Correspondence e-mail: bakary.macy.samatey@gmail.com

Supplementary information

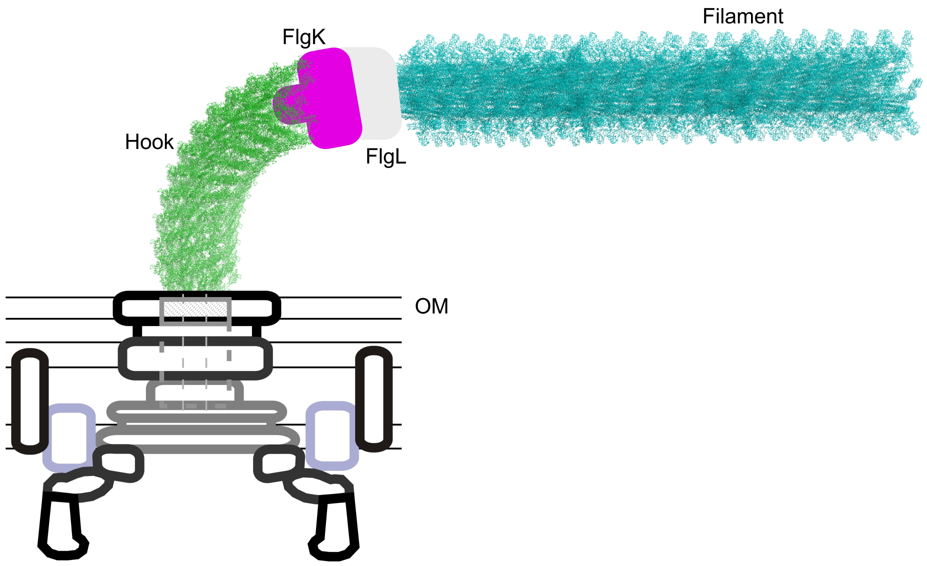

Figure S1. Schematic view of the bacterial flagellum hook and filament showing the Hook-filament junction. View of the bacterial flagellum with a schematic representation of the motor and the hook basal body. The hook (green) and the filament (cyan) are connected by a junction consisting of two rings made of multiple copies of FlgK (purple) and FlgL (grey).

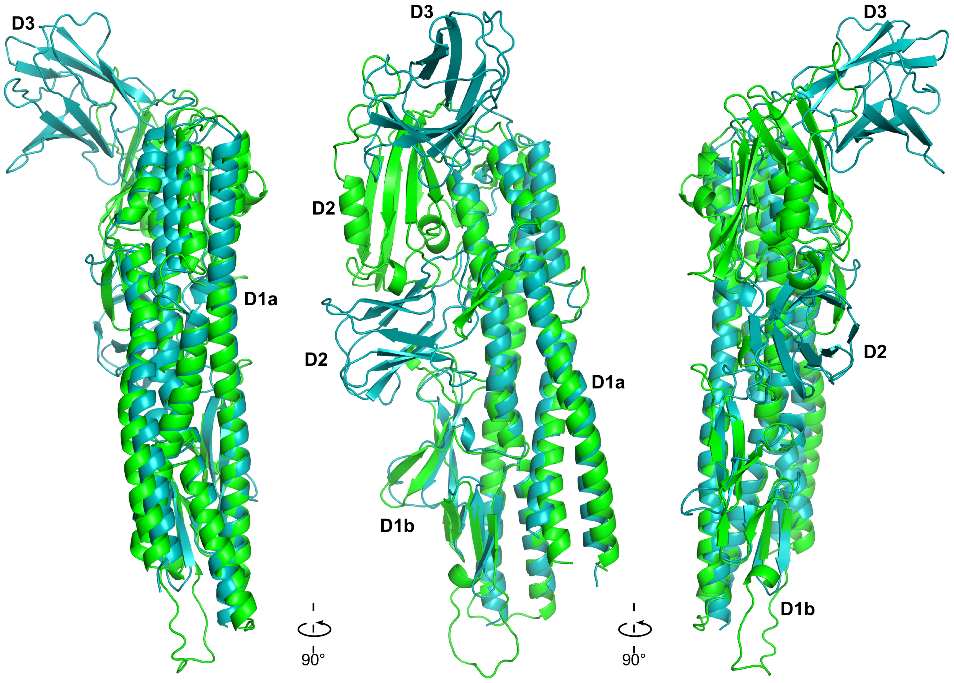

Figure S2. Overall comparison of FlgK structures. Views of the superposition of FlgKcj58 in green and FlgKbp64, in cyan. These structures superimpose with an RMSD of 1.5 Å over 291 residues. These structures have 498 and 551 residues, respectively. Alignments were done with C-alpha Match[39].

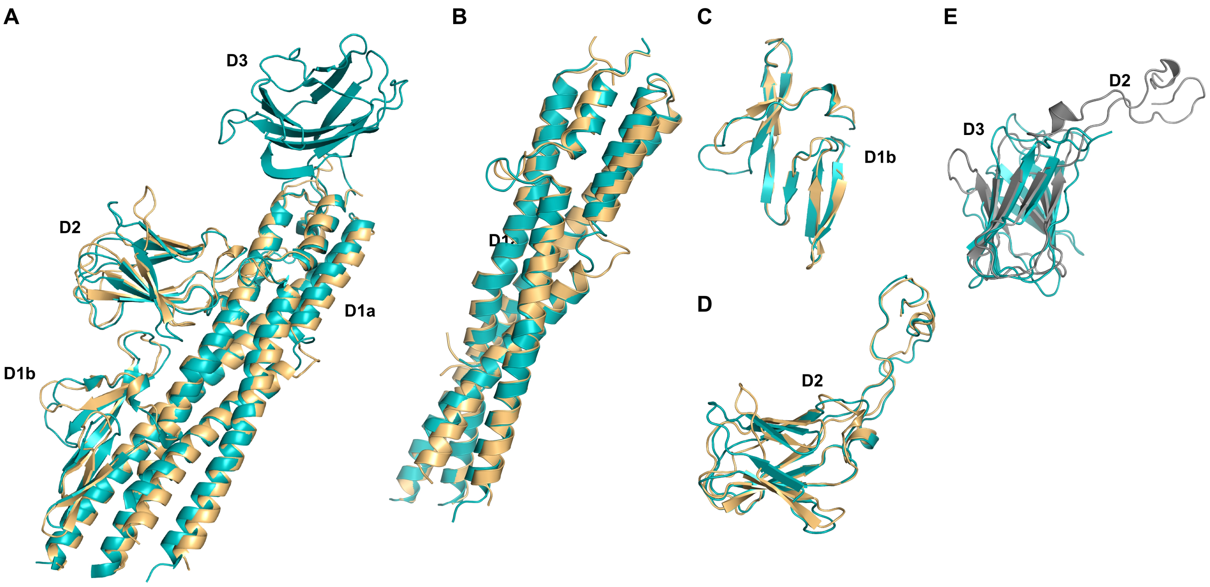

Figure S3. Comparison of FlgKbp64 and FlgKse49. Superposition of the different domains of FlgKbp64 from *B. pseudomallei*, in cyan, and FlgKse49 from *S. enterica*, in gold. (**A**) Overall superposition of FlgKbp64 and FlgKse49. (**B**) Domains D1a superimpose with an RMSD of 1.1 Å over 229 residues. (**C**) Domains D1b superimpose with an RMSD of 1.1 Å over 63 residues. (**D**) Domains D2 superimpose with an RMSD of 1.3 Å over 107 residues. (**E**) Domains D2 in grey and D3in cyan, both from FlgKbp64, superimpose with an RMSD of 1.7 Å over 62 residues. Both domains have 123 and 124 residues, respectively. The alignments were done with C-alpha Match[39].

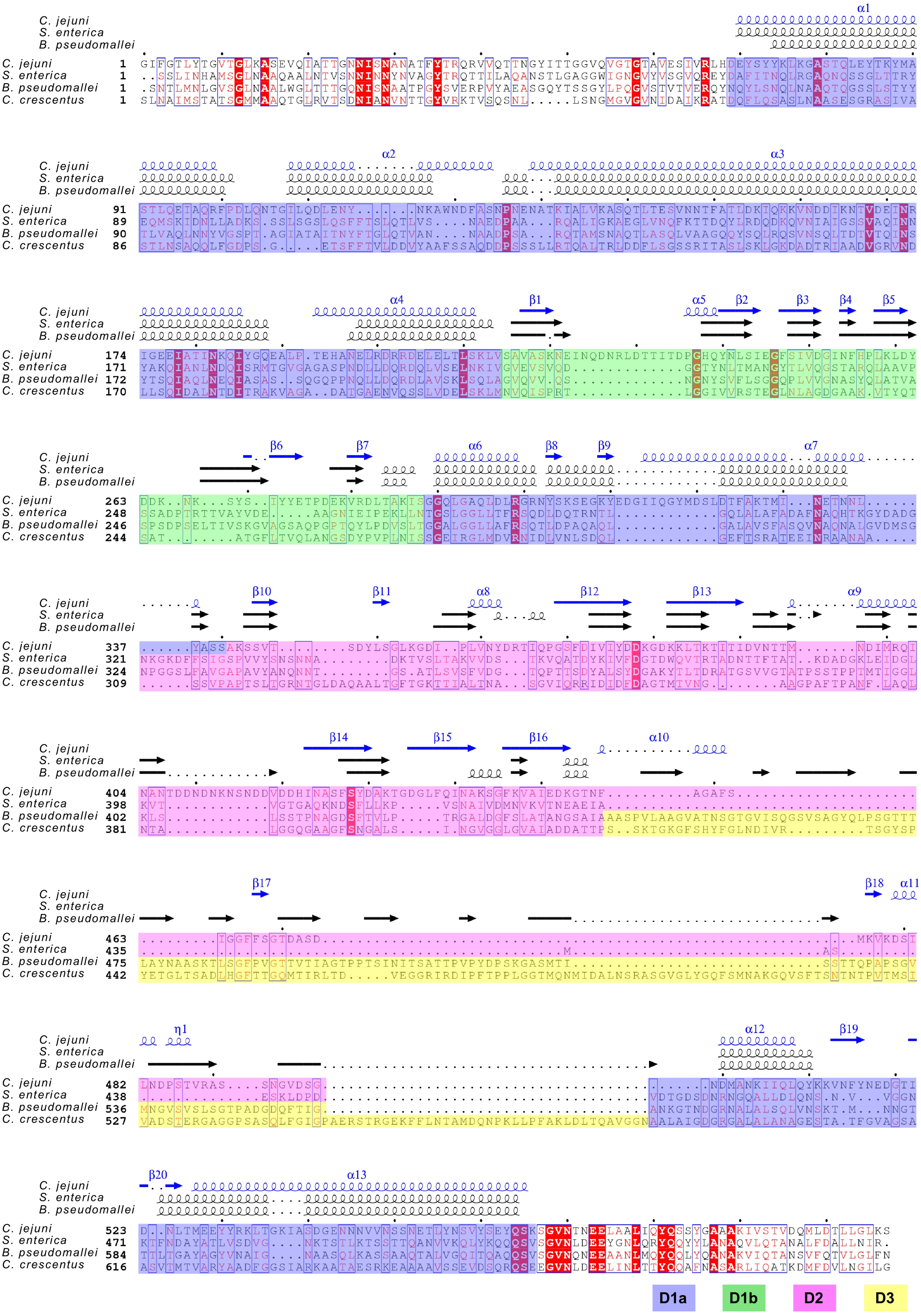
C. jejuni
S. enterica
B. pseudomallei
C. crescentus
D1a
D1b
D2
D3

43

Figure S4. Sequence alignment of FlgK proteins. Sequence alignment of FlgK from C. jejuni, *S. enterica*, *B. pseudomallei* and *C. crescentus*. A representation of the secondary structures of the FlgKcj58 (blue), FlgKse49 and FlgKbp64 are also shown. The structure of domain D0 composed of the N-terminal and the C-terminal chains is not known. The different domains, D1a, D1b, D2 and D3, are colored in grey, blue, green, purple and yellow, respectively. Domain D3 is only found in FlgKbp and FlgKcc. Identical residues are written in white with a red square, while similar residue are written in white. Sequence identity between FlgKcc and FlgKcj, FlgKse and Flgkbp is 19.6%, 22.9% and 23.7%, while the sequence similarity is 32.8%, 36% and 36.9%, respectively. The alignment was done with Clustal Omega[37], and secondary structure rendering was done with ESPript 3.0 (ref. 38).
